# Coping strategies of psychiatrists and psychiatry trainees following patient suicide and suicide attempt: A national cross-sectional study in Saudi Arabia

**DOI:** 10.1371/journal.pone.0300004

**Published:** 2024-03-07

**Authors:** Moayad S. Alawad, Mohammed A. Alammari, Mohannad M. Almanea, Rayan S. Alhumaid, Azzam S. Alkhalifah, Fahad D. Alosaimi

**Affiliations:** 1 Psychiatry Residency Joint Program, Riyadh, Saudi Arabia; 2 College of Medicine, Qassim University, Al-Mulida, Saudi Arabia; 3 Pediatric Residency Program, Maternity and Children Hospital, Buraydah, Saudi Arabia; 4 Department of Psychiatry, College of Medicine, King Saud University, Riyadh, Saudi Arabia; Dilla University College of Health Sciences, ETHIOPIA

## Abstract

A patient’s suicide or suicide attempt is a challenging experience for psychiatrists. This study aimed to explore the common coping strategies and habits developed by psychiatrists/trainees following such incidents. A self-administered questionnaire was distributed among participants in Saudi Arabia. The study enrolled 178 participants, of whom 38.8% experienced a patient’s suicide, 12.9% experienced a patient’s severe suicide attempt, and 48.3% did not encounter any suicidal events. The most frequently utilized sources of support were colleagues (48.9%), team discussions (41.3%), and supervisors (29.3%). Only 21.4% received formal education in coping with a patient’s suicide. Approximately 94.9% reported a lack of support systems within their institution. The study highlighted the coping strategies most commonly employed by psychiatrists/trainees and revealed that the majority of participants reported no changes in their daily habits. The findings underscore the need for a structured support system and formal educational resources to address the existing deficit. Mental health organizations must take action to ensure adequate resources for healthcare providers.

## Introduction

Suicide is considered one of the crucial causes of death globally and a significant health issue [1]. It is a challenging experience for the deceased patient’s family, friends, and psychiatrists [2]. Patient suicide can deeply affect psychiatrists, causing emotional distress, impacting their professional performance, and potentially leading to post-traumatic stress disorder (PTSD) symptoms [2–5]. Studies conducted in the USA, England, Canada, Belgium, and Ireland reported the percentages of psychiatrists who experienced patient suicides (not attempts) to be 50%, 51%, 80%, 82%, and 98%, respectively [4, 6–9]. Furthermore, a systematic review found that the percentage of psychiatric trainees experiencing patient suicide by their patients ranges from 31% to 69% [10]. In 2019, the rate of suicide in Saudi Arabia (SA) per 100,000 population was six, higher than the rate of the Arab world 4.2, but lower than the worldwide rate of 9.2 [11]. A Saudi study review reported that suicide could be under-reported due to religious reasons and the highest rate of suicide was among non-Saudi immigrants [12]. To cope with stress, psychiatrists were found to employ both positive strategies (e.g., seeking support from colleagues, exercising, socializing) and negative strategies (e.g., worrying, carrying on as if everything were fine, losing sleep), as indicated in a study [13]. The utilization of certain negative strategies (e.g., behavioral disengagement, self-blame, denial) was linked to heightened psychological distress [14]. In contrast, specific positive strategies (e.g., problem-solving, receiving support from partners or colleagues, engaging in recreational activities, and taking holidays) were found to be associated with reduced psychological stress [14, 15]. Regarding coping measures after patient suicide, there are two ways to deal with such events: (A) measures before the event and (B) measures post-suicide/postvention. Measures before the event include training guidelines, suicide risk assessment, obtaining data about risk factors, epidemiology, and suicide dynamics [2, 16]. The measures after the event focus on providing support for psychiatrists through case reviewing, conversations with supervisors and colleagues, and tailored training are helpful resources to cope with the aftermath [8]. There is a lack of training regarding post-suicide coping, and few training centers have started to develop a formal support system and suicide postvention kits [17]. However, they seem to be underutilized, and there is a controversy regarding their effectiveness [7, 17, 18]. On a personal level, some health practitioners utilize healthy coping habits like spirituality and exercise. On the other hand, others may tend to smoke or self-medicate [19, 20]. Although this is a global issue, scarce regional studies give the cultural aspects of the Islamic and Arab worlds. Two Saudi studies explained how consultants and trainees of many medical specialties deal with work-related stress [21, 22]. These studies found that high-stress level was associated with maladaptive coping measures, and consultants used religion most frequently [21, 22]. Few studies showed that the impact of exposure to a severe patient suicide attempt was comparable to exposure to patient suicide [5, 23]. However, how psychiatrists/trainees cope with patient’s severe suicide attempts was rarely studied. Therefore, this study aims to identify different coping strategies and habits used by psychiatrists/trainees after a patient’s suicide or suicide attempt in Saudi Arabia. The second goal is to assess post-suicide coping training and the availability of support systems (postvention).

## Materials and methods

This cross-sectional study was carried out between March and August 2020. The study population was pooled out of all psychiatrists registered at the Saudi Commission for Health Specialties (SCFHS), a registry and regulatory body that supervises and certifies all healthcare workers and medical trainees in Saudi Arabia.

### Population

The study included psychiatrists and psychiatric trainees in all regions of Saudi Arabia. All psychiatrists/trainees who are not working in Saudi Arabia were excluded. A psychiatry consultant is a physician who completed a Saudi psychiatry specialty (or equivalent) and practiced for at least three years [24].

### Dependent and independent variables

The independent variables in this study consist of experiencing patient suicide or experiencing a serious suicide attempt by a patient. A serious suicide attempt is defined as an attempt resulting in permanent damage or disability, such as facial disfigurement or fractures. This definition aligns with previous definitions in the literature [23]. The dependent variables include coping measures and changes in daily habits.

### Sampling procedure and sampling techniques

Considering the response rates observed in similar international studies (16.4% among psychiatric trainees) and local studies (5.1% among physicians and 25% among residents), a low response rate was anticipated [21–23]. Therefore, we decided to contact all psychiatrists and trainees registered at SCFHS in order to achieve the desired sample size. The recruitment process for participants involved two stages. During the first stage, we reached out to all psychiatrists/trainees registered on SCFHS’s email list (N = 1007). We repeatedly sent the study questionnaire and written informed consent to these participants over three successive periods. As a result, we received 137 completed questionnaires. In the second stage, to further enhance the response rate, we employed convenience sampling by contacting participants through work-related groups on social media platforms. This approach yielded an additional 55 responses, bringing the total number of participants to 192. Following data collection, 14 participants were excluded due to incomplete questionnaire responses and missing information. All participants were asked about their experience with patient suicide (completed suicide). Participants who reported no exposure to patient suicide were automatically asked about encountering a serious suicide attempt. This approach ensures that both patient suicide and patient serious suicide attempt events are represented in the study.

### Data collection tool

We developed an online self-administered questionnaire based on the literature review (available in supplementary material). A multi-disciplinary committee covering academia, epidemiology, and psychiatry validated the questionnaire’s content. The questionnaire was piloted by participants (N = 20) and modified after the feedback before final distribution. The questionnaire consists of the demographics of psychiatrists, frequency of suicide, patient’s characteristics, the impact of the most distressing suicide experience (psychological impact on the psychiatrist), and psychiatrists’ coping strategies. Much data was obtained from this questionnaire, which took 10 minutes to complete. We addressed emotional reactions and the impact of patient suicide and suicide attempts on psychiatrists/trainees in a separate study [5]. For this current study, we are mainly focusing on coping measures, training, and support systems. For coping measures, participants were asked, “Which of the following support measures were helpful to you in dealing with the situation?”. The question and available answers were primarily derived from previous studies [4, 9, 23]. Additionally, based on a review of the literature on habit changes following stress, participants were asked, “Which of the following habits were used more frequently to deal with the situation?” [20, 25]. We asked about the availability of any formal support system in the workplace and if they received any education/training in coping with suicide or suicide attempts. An optional open-ended question was added, asking the participants about their suggestions for a colleague who experienced patient suicide.

### Data management and analysis plan

Statistical analyses were carried out using Statistical Packages for Software Sciences (SPSS) version 26 Armonk, New York, IBM Corporation. Data are elaborated with numbers (percentages) for all qualitative variables, while median, minimum, maximum, and mean ± standard were used to present all quantitative variables. Between comparisons, the Chi-square test and Fisher Exact test were applied for categorical variables, while the Kruskal Wallis test and Mann-Whitney U test were used for continuous variables. Normality tests were performed using the Shapiro-Wilk test. P-value <0.05 has been accepted as the significant level for all statistical tests.

### Ethical consideration

The study was approved by the institutional review board of the College of Medicine of King Saud University in Riyadh City, Saudi Arabia. An online written consent was obtained before participation.

## Results

We enrolled 178 psychiatrists to evaluate their reactions regarding patient suicide or suicide attempt, with a response rate of 19.1%. Table 1 presents the sociodemographic characteristics of the participants.

**Table 1:** Sociodemographic characteristics and experience of suicidal events.

Study Variables	Overall	Experienced Suicidal event		
		Death	Attempt	No
	N (%)	N (%)	N (%)	N (%)
	^(n = 178)^	^(n = 69)^	^(n = 23)^	^(n = 86)^
Age group				
● <40 years	93 (52.2%)	24 (34.8%)	15 (65.2%)	54 (62.8%)
● ≥40 years	85 (47.8%)	45 (65.2%)	08 (34.8%)	32 (37.2%)
Gender				
● Male	127 (71.3%)	55 (79.7%)	17 (73.9%)	55 (64.0%)
● Female	51 (28.7%)	14 (20.3%)	06 (26.1%)	31 (36.0%)
Nationality				
● Saudi	110 (61.8%)	32 (46.4%)	17 (73.9%)	61 (70.9%)
● Non-Saudi	68 (38.2%)	37 (53.6%)	06 (26.1%)	25 (29.1%)
Role of practice				
● Residents	53 (29.8%)	08 (11.6%)	08 (34.8%)	37 (43.0%)
● Psychiatrists	125 (70.2%)	61 (88.4%)	15 (65.2%)	49 (57.0%)
Years in practice				
● ≤10 years	90 (50.6%)	20 (28.9%)	16 (69.6%)	54 (62.8%)
● >10 years	88 (49.4%)	49 (71.1%)	07 (30.4%)	32 (37.2%)
Psychiatrist subspecialty				
● Child and adolescent	06 (03.4%)	04 (05.8%)	0	02 (02.3%)
● Adult psychiatrist	43 (24.2%)	24 (34.8%)	03 (13.0%)	16 (18.6%)
● Geriatric psychiatrist	03 (01.7%)	02 (02.9%)	(04.3%)	0
● Psychosomatic medicine	08 (04.5%)	02 (02.9%)	01 (04.3%)	05 (05.8%)
● Addiction	11 (06.2%)	07 (10.1%)	01 (04.3%)	03 (03.5%)
● None	101 (56.7%)	25 (36.2%)	17 (73.9%)	59 (68.6%)
● Others	06 (03.4%)	05 (07.2%)	0	01 (01.2%)
Place of work				
● Central region	14 (07.9%)	05 (07.2%)	01 (04.3%)	08 (09.3%)
● Eastern region	23 (12.9%)	14 (20.3%)	02 (08.7%)	07 (08.1%)
● Western region	56 (31.5%)	16 (23.2%)	09 (39.1%)	31 (36.0%)
● Southern region	25 (14.0%)	12 (17.4%)	03 (13.0%)	10 (11.6%)
● Northern region	60 (33.7%)	22 (31.9%)	08 (34.8%)	30 (34.9%)
Workplace				
● Government	149 (83.7%)	56 (81.2%)	20 (87.0%)	73 (84.9%)
● Private	11 (06.2%)	05 (07.2%)	02 (08.7%)	04 (04.7%)
● Both	18 (10.1%)	08 (11.6%)	01 (04.3%)	09 (10.5%)

More than half (52.2%) were in the younger age group (<40 years), and the mean age at the time of the investigation was 38.62 years (SD = 9.917, range 24 to 65 years), with the majority being males (71.3%) and Saudis (61.8%). Psychiatrists (62 consultants and 63 specialists) constituted 70.2%, whereas 29.8% were residents (46 trainees and seven service residents). Almost half of the participants have ten years or less of practice (50.6%). Most participants were working in government hospitals (83.7%).

Patterns of patients’ suicide events experienced by the participants Out of our participants, 69 (38.8%) had experienced patients who committed suicide, 23 (12.9%) had patients who had made serious suicide attempts but had not experienced patient suicide, and the remaining 86 (48.3%) did not encounter any suicidal events. Of those who experienced patient suicide, 61 (88.4%) were psychiatrists, and 8 (11.6%) were residents. Out of 46 trainees, only 3 (6.5%) trainees experienced patient suicide. The mean number of suicide cases per respondent inside and outside Saudi Arabia was 3.3, with a median of 2. However, the mean number of suicide cases per respondent inside only Saudi Arabia was 2.06 (38.2% of participants were not Saudis). When we asked about the time since the last suicide, twenty-five (36.23%) participants experienced a patient’s suicide in less than a year. The prevalence of those who experienced patient suicide was more common among the older age group (≥40 years) (p = 0.001), psychiatrists (p = 0.001), and adult psychiatrists (p<0.001). In comparison, the prevalence of those who experienced suicide attempts was more common among Saudis (p = 0.003) and those who spent ten years or less in practice (p<0.001).

### Support measures

Table 2 shows the most common support measures utilized by participants.

**Table 2:** Support measures used in the aftermath of suicide events.

Support measures	Overall	Death	Attempt	P-value §
	N (%)	N (%)	N (%)	
	(n = 92)	(n = 69)	(n = 23)	
Colleagues	45 (48.9%)	34 (49.3%)	11 (47.8%)	1.000
Discussing/Reviewing the case with the team	38 (41.3%)	33 (47.8%)	05 (21.7%)	**0.031** **
Supervisor	27 (29.3%)	22 (31.9%)	05 (21.7%)	0.435
Religious beliefs and practices	22 (23.9%)	15 (21.7%)	07 (30.4%)	0.574
I did not use any supporting measures	19 (20.7%)	10 (14.5%)	09 (39.1%)	**0.018** **
Contact with the suicidal patients’ family	16 (17.4%)	14 (20.3%)	02 (08.7%)	0.341
Contact with another professional who had followed the suicidal patient formerly	06 (06.5%)	05 (07.2%)	01 (04.3%)	1.000
Relatives	05 (05.4%)	02 (02.9%)	03 (13.0%)	0.098
Contact with the suicidal patients’ friend	04 (04.3%)	04 (05.8%)	0	0.569
My therapist/psychiatrist	01 (01.1%)	01 (01.4%)	0	1.000

§ P-value has been calculated using the Fisher Exact test.

** Significant at p<0.05 level.

None of the participants who experienced an event have taken part in their patients’ burial/funeral services. We asked the participants who did not have any suicide events whether taking part in burial/funeral services would be helpful, and we found that only 2.32% said it would be helpful.

We found some differences between genders regarding which support measures were frequently used. The most support measures used by male participants were seeking support through discussing/reviewing the case with the team (48.6%), colleagues (45.8%), supervisors (33.3%), and religious beliefs and practices (23.6%). On the other hand, females tend to prefer colleagues (60%), followed by religious beliefs and practices (25%), discussing/reviewing the case with the team (15%), or supervisors (15%). More males (48.6%) than females (15%) preferred discussing/reviewing the case with the team (p = 0.007). However, 18.1% of male and 30% of female participants did not use any support measures.

Regarding utilizing different support measures, consultants used more measures than others (p = 0.043). There was no statistical significance (p>0.05) for other characteristics like age group, gender, nationality, psychiatrist subspecialty, years in practice, regions, and workplace.

### Habits

Table 3 shows which habits participants used more frequently to deal with suicide events. Most participants (60.9%) did not change their habits after the event. There was no significant difference (all p>0.05) in habits regarding the type of event (suicide vs. attempt) or the sociodemographic characteristics.

**Table 3:** Habits that were used more frequently to deal with suicide events.

Frequently used habits	Overall	Death	Attempt	P-value §
	N (%)	N (%)	N (%)	
	(n = 92)	(n = 69)	(n = 23)	
I did not use any	56 (60.9%)	42 (60.9%)	14 (60.9%)	1.000
Exercising	11 (12.0%)	07 (10.1%)	04 (17.4%)	0.458
Using social media	11 (12.0%)	08 (11.6%)	03 (13.0%)	1.000
Smoking	10 (10.9%)	05 (07.2%)	05 (21.7%)	0.114
Praying	08 (08.7%)	06 (08.7%)	02 (08.7%)	1.000
Eating	07 (07.6%)	05 (07.2%)	02 (08.7%)	1.000
Sleep	07 (07.6%)	06 (08.7%)	01 (04.3%)	0.675
Isolation	04 (04.3%)	03 (04.3%)	01 (04.3%)	1.000
Using prescription drugs	01 (01.1%)	0	01 (04.3%)	0.250
Substance use	01 (01.1%)	01 (01.4%)	0	1.000
Others	08 (08.7%)	05 (07.2%)	03 (13.0%)	0.408

§ P-value has been calculated using the Fisher Exact test.

** Significant at p<0.05 level.

### Formal education and support system (postvention)

Regarding the availability of a formal support system for professionals after experiencing patient suicide, only (5.1%) of the participants have a support system in their workplace. Furthermore, (21.35%) of participants received formal education\training in coping with patients’ suicidal behaviors.

### Recommendations to deal with patient suicide

Some participants (N = 53) answered an optional open-ended question about the suggestions they would give to a colleague who experienced patient suicide. The answers fall under five categories, with some participants covering two categories in a single response. Approximately 27 (50.9%) answers involved reviewing the case with supervisors, discussing the matter with colleagues, and seeking support or counseling. Another group of participants (N = 11, 20.8%) provided practical advice focused on conducting suicide risk assessment, implementing preventive measures, admitting high-risk patients, and offering psychoeducation to families. Additionally, 11 (20.8%) participants acknowledged the significance of self-reflection, praying, meditation, and considering such events as inevitable learning experiences in the field of psychiatry. Furthermore, 7 (13.2%) participants advocated for the development of adequate training courses and the establishment of a specialized team to provide support and assessment in the aftermath of such events. Finally, 2 (3.8%) responders recommended seeking assistance from legal agencies.

## Discussion

In this study, we expanded the scope and examined how psychiatrists and psychiatric trainees cope with suicide and suicide attempts. We found that participants utilized more support measures when they experienced patient suicide as compared to patient suicide attempt. The study also gave a different geographical and cultural/religious aspect of how participants in Saudi Arabia cope with these events. Furthermore, we explored training roles and the availability of postvention programs.

### Patterns of patients’ suicide events experienced by the participants

Although several international studies have been carried out on the prevalence of patient suicide among psychiatrists, surprisingly and up to our knowledge, no Saudi national studies have been done in this regard. In our study, the overall rate of suicide prevalence among psychiatrists and psychiatric trainees is 38.8%, which is lower than in other countries. For example, the prevalence rate in the US is 63.6% [26]. Furthermore, the prevalence rate in our study among psychiatrists (48.8%) was lower than studies in the US (51–76.1%), the UK (80%), Germany (88.4%), and Belgium (91.6%) [4, 7, 9, 26, 27]. Also, the prevalence rate among Saudi psychiatric trainees (6.5%) is lower than colleagues in 7 Western countries, as shown in a recent review where the average prevalence was 46.4% (range from 12% to 69%) [28]. The lower rates in Saudi Arabia can be partially explained by the low suicide mortality rate per 100,000 population compared to other countries [11]. Another possible explanation could be the fragmented care (lack of coordination between healthcare organizations) which makes psychiatrists in Saudi Arabia unaware of such suicide events of their patients [29]. Moreover, Saudis tend to disapprove of suicidal disclosure and believe that suicidal behaviors should be hidden [30]. This might lead to the underreporting of suicides.

### Support measures

Talking to a colleague after a patient’s suicide/attempt was the most frequently used coping method (48.9%) for dealing with these stressful events. This finding aligns with previous studies and is considered the most helpful approach as it allows for the sharing of emotions with someone who understands [8, 31]. Moreover, recognizing that suicide is neither a personal failure nor a unique event can help reduce the sense of isolation [6]. In our study, discussing the case with the team was the second most common measure, followed by discussing the case with the supervisor. Although supervision is a commonly employed method, it appears to be less effective than talking to colleagues [8, 32]. Previous studies have suggested that the team should review the case in a friendly and educational manner rather than engaging in a judgmental inquisitional session targeting a single psychiatrist [6]. Religious beliefs and practices served as a prominent support measure (23.9%), following supervision. This finding can be attributed to the fact that our study was conducted in an Islamic country, where religion plays a prominent role in coping strategies. This aligns with previous studies conducted in Saudi Arabia, where religion was identified as the most frequently used adaptive coping strategy among physicians [21, 22]. In contrast to studies conducted in Western countries, where 15–26% of psychiatrists attended the funeral of their deceased patients, none of the participants in our study reported attending the funeral [9, 31, 33, 34]. There are several possible reasons for this disparity. One explanation could be that some of the participants were not aware of the exact timing of the patient’s death due to a fragmented healthcare system (lack of coordination between healthcare organizations and limited patient-doctor communication channels) [29]. Religious and social reasons also play a significant role in this context. For example, Muslim women (including female psychiatrists) rarely attend the burials and they are not expected to attend according to Islamic customs [35]. Moreover, suicide is considered a major sinful act in Islam [36]. Families tend to conceal suicide because of shame and stigma and might get ostracized by the community and viewed with suspicion [37]. These cultural considerations contribute to suicides being under-reported in Islamic countries to the extent that some families deal with private hospitals to avoid registration and reporting such cases as suicide in Pakistan [37]. It is important to note that attending a deceased patient’s funeral presents an ethical dilemma, and there is no clear consensus on appropriate therapeutic conduct after patient’s death [38]. Attending such funerals carries the risk of breaching confidentiality and blurring professional boundaries, requiring therapists to exercise caution in avoiding the disclosure of sensitive information [39]. This careful approach may present challenges for therapists in fully expressing their grief, potentially leading to a sense of disenfranchisement [39, 40]. Finally, around 39.1% of participants who experienced patient suicide attempts did not use any support measures compared to 14.5% of participants who experienced patient suicide. This might be because complete suicide had more impact on the participants, as suggested by Scocco and colleagues [3]. However, a recent study among psychiatric trainees reported that exposure to a patient suicide attempt could be as distressing as exposure to a patient suicide [23].

### Habits

In our study, most participants (60.9%) did not have changes in their habits after the events. Probably due to utilizing other support measures such as colleagues. Exercising and social media use, followed by smoking, were the most used habits. A similar study found that social workers mainly used exercise as a coping habit after experiencing a suicidal event [20]. Generally, physicians tend to deal with stress and burnout by exercising to improve their mood [41, 42]. Negative habits like smoking have been used as a stress reliever (11%). Negative habits might increase if positive habits are not utilized [43].

### Formal education and support system (postvention)

Unfortunately, in our study, only 21.35% of the participants reported the existence of formal education inside their institutions. Similarly, one Canadian study involving 197 psychiatric residents in postgraduate years found that only 32% of them received education about the impact of suicide on them in their careers [44]. Another study in the United States involving 106 psychiatric programs found that postvention was taught by only one-quarter of programs, and 19% of the respondents felt prepared for the aftermath of patient suicide [45]. Two studies found that suicide educational programs can increase the knowledge and awareness of support systems to deal with patient suicide [46, 47]. However, whether these programs will be helpful in the aftermath of actual suicide events is unknown. Most participants (94.9%) reported a lack of a formal support system (postvention) in their institutions. Similarly, only 21% of psychiatry programs in the USA reported having a written postvention protocol [18]. One study highlighted that less than 1 in 10 implemented an evidence-based intervention in their facility [17]. Flemish research found that less than half of responders reported the existence of support systems in their facility [9]. The lack of a support system may have a negative effect, especially for psychiatric trainees. The authorities should note that this issue affects doctors in many domains, emotionally and professionally. Brown states that establishing policies by residency programs and the department of psychiatry to deal with complex patients is very important and will make trainees feel prepared to deal with serious events [2].

### Limitations

The primary limitations include being a retrospective study, which may inherently have a recall bias for some variables. Prospective validation of the findings would be desirable, but it would take years to develop an adequate sample size and be hugely expensive. The overall response rate was slightly low, which could be attributed to a relatively lengthy questionnaire. Furthermore, there is no guarantee that all emails sent reached the intended population (i.e., some emails did not appear to them and were considered spam emails). Moreover, previous international similar study and local studies among physicians and trainees have shown similar low response rates [21–23]. Additionally, female participants were fewer than males, which could affect the generalization of the female gender. There might be a volunteer bias because participants who had a suicide would be more likely to fill out the questionnaire, resulting in overestimating the results. Studying the relationship between the psychological impact and the way of coping with the trauma is advised for future studies. Although the study questionnaire was derived mainly from previous studies and has been face-validated by the authors, future studies might use more validated scales (e.g., the Brief Cope and the Professional Practice Impact Scale).

## Conclusions

The findings of our study showed that the most utilized helpful coping strategies among psychiatrists/trainees who experienced suicidal events were seeking help from colleagues, discussing with the team, and supervision. The study also explored cultural and religious aspects of coping with these events. Most participants did not change their habits to deal with these events. However, there was an increase of using social media, exercise, and smoking. The study showed a lack of formal education in dealing with suicide and almost no presence of formal postvention measures in the workplace. Since it is impossible to prevent all patient suicides, the study confirms the need for further understanding of helpful interventions to mitigate the impact on psychiatrists/trainees.

## Supporting information

S1 Data (XLSX)
